# Dexmedetomidine as Part of a Multimodal Analgesic Treatment Regimen for Opioid Induced Hyperalgesia in a Patient with Significant Opioid Tolerance

**DOI:** 10.1155/2017/9876306

**Published:** 2017-09-25

**Authors:** Richard K. Patch III, Jason S. Eldrige, Susan M. Moeschler, Matthew J. Pingree

**Affiliations:** ^1^Division of Critical Care Medicine, Department of Anesthesiology & Perioperative Medicine and Division of Pulmonary and Critical Care Medicine, Department of Medicine, Mayo Clinic, Rochester, MN, USA; ^2^Division of Pain Medicine, Department of Anesthesiology & Perioperative Medicine, Mayo Clinic, Rochester, MN, USA; ^3^Department of Physical Medicine and Rehabilitation, Mayo Clinic, Rochester, MN, USA

## Abstract

Acute postoperative pain in patients with opioid tolerance creates a significant management challenge for anesthesiologists and pain medicine physicians. A multimodal approach is key; however other factors can complicate management such as opioid induced hyperalgesia. We present the case of a patient on large amounts of intrathecal opioids for chronic pain syndrome with opioid induced hyperalgesia after an exploratory laparotomy. Dexmedetomidine was utilized successfully as part of a controlled multimodal analgesic plan and should be a consideration for opioid tolerant patients experiencing opioid induced hyperalgesia.

## 1. Introduction

Chronic pain, as defined by the American Society of Anesthesiologists, is pain not directly related to neoplastic involvement extending in duration beyond the expected temporal boundary of injury [1]. The International Association for the Study of Pain (IASP) further defines chronicity as pain which is present for at least three months duration or longer [2]. According to a recent Institute of Medicine (IOM) report, over 100 million Americans suffer from chronic pain; this is a leading cause of disability and represents more US patients than those with cardiovascular disease [3–5]. Opioids are one option for the treatment of chronic pain, yet their effectiveness as a long-term therapeutic option has not been established [6, 7]. Chronic pain patients on large amounts of opioids represent a management conundrum for anesthesiologists, primary care providers, and pain medicine physicians in the outpatient and perioperative setting [6, 7]. Given the extreme tolerance to opioid medications that may be exhibited in this population, multimodal analgesia is of the utmost importance.

Opioid induced hyperalgesia (OIH) is defined as a state of enhanced nociceptive sensitization due to exposure to opioids [8]. Opioid induced hyperalgesia is not located at the site of injury and is typically diffuse and ill defined. No established diagnostic criteria exist to diagnose OIH. It is suspected when pain is perceived to increase with increasing opioid use [9]. Opioid induced hyperalgesia can occur in the acute setting and one does not need to be receiving chronic opioids for it to occur. The pathophysiology of OIH has yet to be completely elucidated. One possibility is activation of descending pain pathways from the medulla resulting in certain neurons uniquely responding to opioids [9]. Another possibility is alteration of the central glutaminergic system and the excitatory NMDA neurotransmitter [8]. Regardless of the mechanism OIH in patients on chronic opioids complicates their management.

Dexmedetomidine, a highly selective *α*-2 agonist, may be an increasingly important adjunct to consider within the multimodal approach to postoperative pain management. Dexmedetomidine is a potent anxiolytic, while also providing opioid-sparing analgesic effects [10]. The mechanism of antinociception has not been completely elucidated, but it is believed that it is secondary to *α*-2 receptor stimulation in the central nervous system and spinal cord. We present a case of a patient with chronic pain on large amounts of intrathecal narcotics who developed OIH after abdominal surgery in which dexmedetomidine was utilized as part of a multimodal analgesic regimen.

## 2. Case Description

A 55-year-old female presented for a laparotomy with abdominal exploration, small bowel resection, and lysis of adhesions secondary to sclerosing mesenteritis. The patient had a history of chronic pain syndrome. In 1997 she had an intrathecal drug delivery device implanted to treat refractory chronic back pain in the context of four prior lumbar laminectomies and fusion at an outside medical facility. Her chronic abdominal pain began in 2009 after a small bowel obstruction required an exploratory laparotomy, at which time fibrotic strictures were found which required small bowel resection at the ileum. The patient's pain continued and she went on to undergo cholecystectomy in 2010. In 2011 she underwent evaluation by a local gastroenterologist, with upper endoscopy revealing signs of bile reflux gastritis and retained food. Concomitant motility testing revealed gastroparesis and she was ultimately diagnosed with narcotic-related gastroparesis. In an attempt to decrease the amount of opioids she was receiving from her intrathecal device, she had a spinal cord stimulator (SCS) placed as a therapeutic adjunct. Her pain was marginally improved with the stimulator, though this device ultimately failed and stopped working. Despite the SCS failure, the patient elected not to have the stimulator explanted. Throughout this time course, the patient was also treated concurrently with multiple medication regimens including gabapentin, long acting morphine, fluoxetine, and benzodiazepines.

In 2013, due to continued abdominal pain and increasing intrathecal opioid requirements, a repeat CT scan of the abdomen showed enhancement of the terminal ileum and rectosigmoid colon with a new mass-like area of mesenteric inflammation. The diagnosis of sclerosing mesenteritis was considered and she presented to our institution for further evaluation and management.

During preoperative evaluation, the patient stated her abdominal pain was constant, 24 hours a day, seven days a week. Utilizing the Agency for Healthcare Research and Quality's numeric pain intensity scale (NPIS) her pain was always an eight out of ten. Additionally, it was exacerbated with food, alcohol, the cold, and stress. She noted that she went to sleep and awoke with the same level of pain. Her current medication regimen included trazadone 200 mg orally at night and probiotics. The intrathecal drug delivery system (IDDS) included fentanyl (25,000 mcg/mL) and Bupivacaine (15 mg/mL), infusing at continuous infusion doses of 6000 mcg and 8 mg per day, respectively. Additionally, there were 11 pre-set boluses for fentanyl at 1200 mcg each (given over three-minute durations) per day. In summation, the patient was receiving a total daily dose of 19,702 mcg of fentanyl intrathecally when accounting for both continuous infusion and bolus dosing; this correlates with an oral morphine equivalent (OME) of 591 grams per day. This amount was calculated using a 100 : 1 conversion between intravenous and intrathecal fentanyl, a 100 greater fold potency of fentanyl, and a 3 : 1 conversion between oral and intravenous morphine.

Preoperatively, regional analgesia with an epidural was extensively discussed with the patient; however given her multiple back surgeries and chronic back pain, she declined. As previously mentioned, the patient was treated with gabapentin in the past without any benefit and, as such, she declined preoperative administration. Her operative course was unremarkable. Anesthesia was induced with propofol and succinylcholine, endotracheal intubation was uneventful, and maintenance was provided with isoflurane in air and oxygen. Intraoperative opioids were rotated to hydromorphone totaling 15 mg and additional analgesia consisted of a ketamine infusion of 0.2 mg/kg/hr, acetaminophen 1000 mg IV, ketorolac 15 mg IV, and local infiltration of the wound with 20 cc of liposomal bupivacaine. Antiemetics included droperidol, ondansetron, and dexamethasone while ciprofloxacin was administered for surgical site prophylaxis due to a penicillin allergy. Her hemodynamics were satisfactory throughout the case and she was extubated at the end and transferred to the postanesthetic care unit (PACU) for recovery.

In the PACU the patient's pain was extremely difficult to control. She received escalating boluses of hydromorphone totalling an additional 6 mg and 40 mg of ketamine in addition to an increase in her ketamine infusion to 0.3 mg/kg/hr. The inpatient pain service, who manages both acute and chronic pain, was contacted to assist with management. The patient was hypertensive and tachycardic with a normal temperature, elevated respiratory rate, and oxygen saturations of 98% on 2 L nasal cannula. Examination revealed an unremarkable cardiopulmonary exam with a soft but diffusely tender abdomen to palpation. She noted her pain was progressively becoming worse over the course of her PACU stay and was some of the worst pain she had ever experienced. Her hemoglobin was 10 g/dL down from 11.7 g/dL preoperatively; electrolytes and coagulation profile were all within normal limits. After discussion with the surgical service, no intra-abdominal process was felt to be the etiology and she was diagnosed with an acute pain crisis complicated by OIH. Given the use of high dose intrathecal opioids, substantial ketamine infusion, and current multimodal regimen, we elected to initiate a dexmedetomidine infusion. Per institutional protocol, she was transferred to the progressive care unit (PCU) for monitoring during this infusion.

While in the PCU her pain continued to be difficult to control. She required a dexmedetomidine infusion of 0.8 mcg/kg/hr, ketamine infusion of 0.7 mg/kg/hr with 10 mg IV boluses every 30 minutes as needed, lidocaine patches around her incision, an additional dose of ketorolac, and scheduled acetaminophen. A narcotic patient controlled analgesia (PCA) system was not prescribed given signs and symptoms of OIH. Boluses of 1 mg of hydromorphone every 30 minutes as needed were available and she had continued use of her personal therapy manager (PTM) to provide the previously prescribed intrathecal fentanyl boluses. This regimen provided adequate postoperative analgesia. Additionally, the patient was found to be hemodynamically stable throughout her admission without any overt signs of pain-sedation mismatch.

Over the course of next 48 hrs the dexmedetomidine and ketamine infusions were weaned while the interval of IV boluses of hydromorphone and ketamine was progressively increased to every four hours. On postoperative day three she was transferred to the floor and transitioned from intravenous opioids to oral hydromorphone. At the request of the patient, oral medications were chosen rather than making adjustments to the IDDS. Throughout her hospitalization she experienced no opioid related adverse events such has constipation or sedation. She was discharged on hospital day seven with a prescription of hydromorphone 4 mg orally as needed every 6 hrs and plans to follow-up with her primary pain physician. At her three-month follow-up, her chronic abdominal pain was noted to be better and her intrathecal fentanyl usage had been reduced to 11,000 mcg per day. Furthermore, she was to be evaluated by a pain rehabilitation center at home to address her ongoing pain syndrome.

## 3. Discussion

Chronic pain continues to be one of the most common presenting complaints in the primary care setting. Opioids are an option for nonmalignant pain though chronic use remains controversial, with a general paucity of evidence for long-term benefit [6]. As a result of chronic use, however, patients often develop clinically significant opioid tolerance and opioid induced hyperalgesia. Dexmedetomidine may offer distinct clinical advantages in this patient population, due to opioid-sparing effects as well as anxiolysis. Interestingly, previously published work by Belgrade and Hall also suggests that dexmedetomidine may help physiologically reset opioid sensitivity in otherwise tolerant individuals and in patients suffering from OIH [11]. The precise mechanism of antinociception has not been fully elucidated, but it is *α*-2 receptor stimulation in the central nervous system and spinal cord is thought to play a vital role [10].

Perioperatively, data for the use of dexmedetomidine as part of a multimodal analgesic regimen is becoming well established. A meta-analysis in 2012 of close to 1800 patients, of which 339 received dexmedetomidine, revealed that systemic *α*-2 agonists decreased postoperative opioid consumption and pain intensity [12]. Additional trials have shown that the addition of dexmedetomidine to a sufentanil infusion achieved better analgesic effect and greater patient satisfaction in patients undergoing abdominal hysterectomy for 72 hours postoperatively [13]. Intraoperative infusions of dexmedetomidine have also shown that postoperative pain scores and Ramsay Sedation Scale scores were lower and morphine consumption was lower during the first 24 hrs after surgery [14]. When dexmedetomidine was added to propofol and remifentanil in patients undergoing an abdominal colectomy, they had lower visual analog pain scores and consumed less morphine compared to the addition of saline to propofol and remifentanil [15]. Moreover, pain scores were lower at rest for the first 48 hrs after laparoscopic colorectal surgery; however this group had no difference in morphine utilization [16]. An additional meta-analysis in 2013 examined randomized controlled trials of postoperative pain control utilizing dexmedetomidine compared to placebo at one, two, four, 24, and 48 hours postoperatively [17]. Nine of the trials examined postoperative pain scores revealing that patients receiving dexmedetomidine had lower pain scores at all time points. It is noteworthy that while the mean difference in pain scores remained statistically significant, the means decreased from −1.59 at one hour to −0.41 at 48 hours. Patients receiving dexmedetomidine also had lower morphine consumption at one, two, four, 24, and 48 hours postoperatively [17]. Fourteen of the trials showed significantly higher intraoperative bradycardia in dexmedetomidine treated patients with a relative risk (RR) of 2.66 and a number needed to harm (NNH) of 6.25. The dexmedetomidine group also had higher rates of postoperative bradycardia; however this result failed to show any significance [17].

Less well established is the role of dexmedetomidine for treatment of OIH. In a small retrospective case series 11 patients with OIH and intractable pain were given a dexmedetomidine infusion. Seven of the eleven patients had substantial reductions in their baseline opioid requirements [11]. Patients undergoing laparoscopically assisted vaginal hysterectomy with remifentanil-induced hyperalgesia from an infusion of 0.3 *μ*g/kg/min were randomized to a dexmedetomidine infusion or saline. The group that did not receive dexmedetomidine had a decreased mechanical hyperalgesia threshold, increased pain intensity at one, six, 12, and 24 hrs [18]. Additionally, the morphine consumption was significantly higher leading the authors to conclude that dexmedetomidine reduced the hyperalgesic effect of remifentanil and may have a role in OIH. In a case series of recurrent vasoocclusive episodes in three adolescents with sickle-cell disease who exhibited features consistent with OIH, dexmedetomidine was shown to decrease opioid consumption and pain scores [19].

Ketamine has also been a long established adjuvant both for intraoperative and for postoperative analgesia, specifically in opioid tolerant patients [20]. It played a key role in the management of our patient's postoperative pain as it allowed us to reduce opioids that could exacerbate OIH, while still providing primary analgesic effects itself. Ketamine exerts its analgesic properties by antagonism of the n-methyl-d-aspartate (NMDA) receptor thus reducing presynaptic release of glutamate [21]. This mechanism also allows reduction of central sensitization and the windup phenomena in chronic pain. Low dose ketamine infusions are also a useful adjunct in opioid tolerant patients experiencing acute pain. In opioid dependent patients undergoing spine surgery, ketamine infusions reduced opioid requirements in the first 48 hrs after surgery [22]. The combination of ketamine and dexmedetomidine for acute pain in a patient with chronic regional pain syndrome type I (CRPS-1) was reported to provide a synergistic effect without sequela [23].

Interestingly, our patient received wound infiltration by the surgical team instead of application of a more specific regional anesthetic technique. While neuraxial blocks were discussed and considered (epidural versus paravertebral block/catheter), there was legitimate clinical concern for aberrant anatomy and/or disrupting the indwelling intrathecal system due to a known history of several prior back surgeries and the absence of precise outside medical records to clarify IDDS implantation technique. Other peripheral blocks, such as transversus abdominus plane (TAP) block, could have been placed but the expectation was that such a block would not help the significant visceral pain component expected after exploratory laparotomy. Additionally, it should be noted that there is randomized clinical trial data suggesting that surgical wound infiltration is as effective at pain control as epidural or TAP block interventions [24, 25].

We recognize that other preoperative strategies, such as weaning intrathecal opioids, could have been employed as a viable therapeutic path to attenuate concerns of opioid tolerance and OIH. However, in our case, there were at least two relevant barriers to this clinical consideration. First, the patient was being managed by an out-of-state pain physician who had very different ideas on appropriate medical management of the patient's pain. Intrathecal doses of such magnitude, which exceed reasonable limits suggested by national guidelines such as the Polyanalgesic Consensus Conference (PACC), imply a general unwillingness by both patient and provider to vastly restrict opioid dosing (as would be required) [26]. Additionally, even if an overt willingness to wean had been encountered, both distance and time presented significant barriers. Distance and location otherwise complicated the sort of long-term longitudinal care and follow-up that would be required to wean such high intrathecal doses. The length of time required to significantly wean such high doses may also have presented a moral dilemma; though the surgery was not emergent, the lysis of adhesions for progressive sclerosing mesenteritis presented a clear palliative medical need that could not be easily ignored [22].

Perioperative lidocaine infusion is another adjunct used for postoperative pain that could have been utilized in our patient. However, a systemic review concluded that currently the evidence is low to moderate that the intervention will reduce pain in early postoperative phase, one to four hours, and there is no evidence that that it will reduce pain at 48 hours [27]. Additionally, data on lidocaine infusions in patients with intrathecal drug delivery devices is limited and, given the significant amount of intrathecal opioids the patient was receiving, their effect on her postoperative pain and OIH would likely have been minimal.

## 4. Conclusion

In conclusion, we present the case of OIH in a patient with an intrathecal drug delivery device receiving an oral morphine equivalent of 591 gms of intrathecal fentanyl that required an aggressive multimodal postoperative analgesic regimen after exploratory laparotomy. Dexmedetomidine was utilized as part of that regimen with safe and effective results. Our patient was monitored in a PCU setting and there were no hemodynamic sequela. We propose that dexmedetomidine is a useful adjunct for the management of OIH, particularly in a patient who is significantly opioid tolerant with high opioid requirements.
